# Impact of updated trial data on the cost-effectiveness of percutaneous mitral repair

**DOI:** 10.1371/journal.pone.0280554

**Published:** 2023-01-26

**Authors:** Martin Connock, Peter Auguste, Jean-François Obadia, Lazaros Andronis, Xavier Armoiry

**Affiliations:** 1 Warwick Medical School, University of Warwick, Coventry, United Kingdom; 2 Chirurgie Cardio-Vasculaire et Transplantation Cardiaque, Hôpital Cardiovasculaire Louis Pradel, Hospices Civils de Lyon and Claude Bernard University, Lyon, France; 3 Pharmacy Department, School of Pharmacy (ISPB) / UMR CNRS 5510 MATEIS / Edouard Herriot Hospital, University of Lyon, Lyon, France; URCEco Ile de France Hopital de l’Hotel Dieu, FRANCE

## Abstract

When updated clinical trial data becomes available reassessing the cost-effectiveness of technologies may modify estimates and influence decision-making. We investigated the impact of updated trial outcomes on the cost-effectiveness of percutaneous mitral repair (PR) for secondary mitral regurgitation. We updated our previous three-state time-varying Markov model to assess the cost-effectiveness of PR + guideline directed medical treatment (GDMT) versus GDMT alone. Key clinical inputs (overall survival (OS) and heart failure hospitalisations (HFH)) were obtained using the 3-year trial findings from the COAPT (Cardiovascular Outcomes Assessment of the MitraClip Percutaneous Therapy) RCT. We calculated incremental cost-effectiveness ratios (ICER) and report how these differ between analyses based on early (2-year) and updated (3-year) evidence. Updated trial data showed an increase in mortality in the intervention arm between two and three years follow-up that was not seen in the control arm. Deterministic and multivariate cost-effectiveness modelling yielded incremental cost effectiveness ratios ICERs of €38,123 and €31,227 /QALY. Compared to our 2-year based estimate (€21,918 / QALY) these results imply an approximate 1.5-fold increase in ICER. The availability of updated survival analyses from the COAPT pivotal trial suggests previous estimates based on 2-year trial findings were over optimistic for the intervention.

## 1 Introduction

Percutaneous repair (PR) with the Abbott Vascular MitraClip system has been developed with the aim of improving clinical outcomes in patients with mitral regurgitation (MR) [1] in which the mitral valve fails to close tightly allowing blood to flow backward from left ventricle into the left atrium potentially leading to heart failure, fatigue, shortness of breath and reduced quality of life. PR is a promising intervention for those patients judged ineligible for or at high-risk from surgery, and represents a less invasive procedure for those for whom surgery could be an option. The efficacy and safety of the MitraClip system in functional or secondary MR (SMR) where there is no organic lesion of the valve that has led to MR has been demonstrated in the COAPT study, a large industry sponsored randomised control trial carried out in the US evaluating clinical outcomes after PR + Guideline-Directed Medical Treatment (GDMT) versus GDMT alone [2]. Two year results from COAPT were published in 2018 [2] and three year results made available in 2019 [3] and in 2021 [4]; four year results as yet have not been published (July 2022), per-protocol results are expected to five years (https://clinicaltrials.gov/ct2/show/NCT01626079).

COAPT data have been used in six cost-effectiveness (CE) analyses in publications spanning 2019 to 2022 [5–10] in studies that use a lifetime horizon (~30 years) so as to accommodate decision-makers’ stipulation that analysis should fully capture benefits and harms. In estimating major benefits such as life years gained (LYG) mortality estimates need to span up to 30 years necessitating considerable extrapolation beyond in-trial observed mortality. Most published CE studies [5–10] make use of 2-year mortality data from COAPT, and one [11] employed 3-year mortality for the GDMT arm to generate mortality in the MitraClip arm by applying a hazard ratio (HR). The inherent uncertainty in these life time models of mortality is reflected in the reported gains in life years that exhibit an almost two fold variation; considerable uncertainty is also reflected when LYG is adjusted according to quality of life estimates.

The 2-year COAPT trial findings [2] reported 12 month and 24 month mortality in the PR + GDMT arm of 19.1% and 29.1% equating to a crude rate of 10% over year 1 to 2. The three-year findings from COAPT [4] showed 19%, 28.2% and 42.8% mortality at years one, two and three for the PR + GDMT arm, equating to crude rates of 9% for year 1 to 2 and of 14.6% for year 2 to 3, a substantial increase (of ~62%) in year two to three over that for the previous year one to 2. These reported mortality rates suggest that CE analysis using three-year data for the MitraClip arm is required. Since such analysis has not yet been undertaken we assessed the potential impact of three-year trial data on cost-effectiveness estimates of PR with MitraClip.

## 2 Materials and methods

We evaluated the cost-effectiveness of the Mitraclip system in SMR patients using our previously published model structure [6] comprising three mutually exclusive health states: alive and free of heart failure hospitalisation, alive with heart failure hospitalisation, and dead. Our focus was on the potential impact of the updated 3-year all-cause mortality results from the COAPT trial. The structure of the economic model, the analysis perspective, annual discount of costs and benefits, time horizon, economic and utility inputs were as described in our previous analysis [6] that was based on the 2-year results from COAPT (Stone et al. [2]). Model output was assessed for sensitivity to inputs using univariate analysis varying survival according to 95% confidence intervals, changing utility in live states by ±10% and costs by ± 20%. Results are presented in a Tornado plot.

We investigated model uncertainty using multivariate sensitivity analysis and bootstrapping with 500 iterations (Gallacher and Achana [12]): overall survival in each arm was increased or decreased according to 95% (CI); costs in each arm varied by ± 20% and utility in the live state in each arm changed by ±10% while utility decrement for HFH was kept constant; these values are in line with those used in other analyses [6, 7, 10]. The results were plotted on the cost-effectiveness plane with 95% CI ellipses as described by Alexandersson et al. [13].

Published graphs were digitised using Digitizelt [14]. Reconstructed individual patient data was obtained using the method of Guyot et al. [15]. Parametric survival models were generated using the *streg* command and the *stgenreg* [16] and the *stpm2* [17] packages in STATA versions 15.0 or later (Statacorp, College Station, TX, USA). Sources of survival (mortality) data and of Heart Failure Hospitalisation (HFH) were taken from reports of the 3-year findings from COAPT by Mack et al. 2019 [3] and 2021 [4].

## 3 Results

### 3.1 Survival

**Fig 1:**
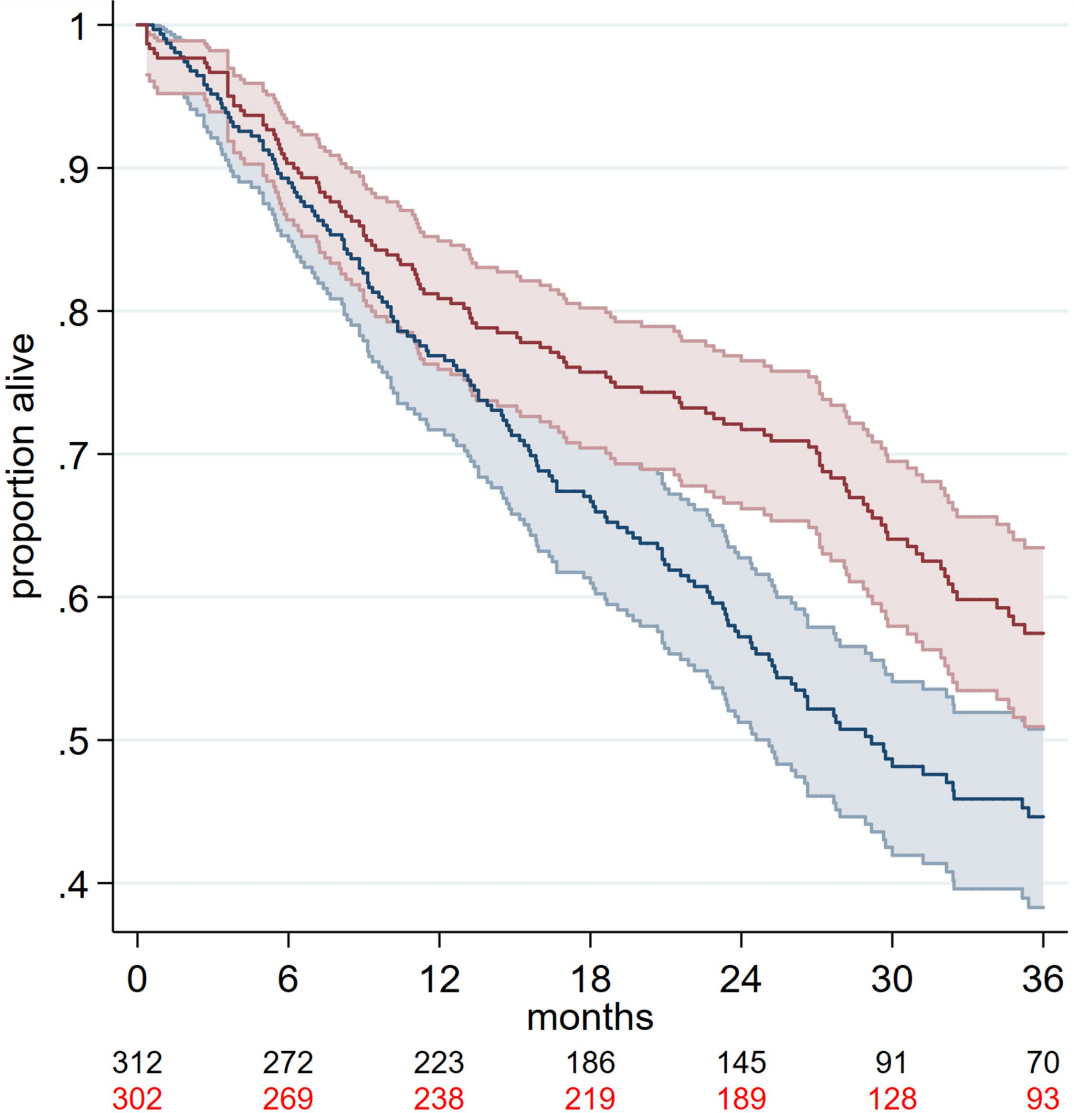
Reconstructed KM plots with 95% CIs for 3-year survival in COAPT. Red represents the PR+GDMT arm and black represents GDMT arm.

Fig 1 shows the reconstructed overall survival KM plot for each arm of COAPT at three years follow-up. The PR+GDMT arm exhibits clearly superior survival compared to the GDMT arm; the intervention arm is characterised by a gradually decreasing trajectory to about 26 months followed by a distinct downturn in survival trajectory to 36 months (S1 Fig in S1 File). In contrast, the plot for the GDMT arm exhibits a steady almost linear trajectory across all three years.

**Fig 2:**
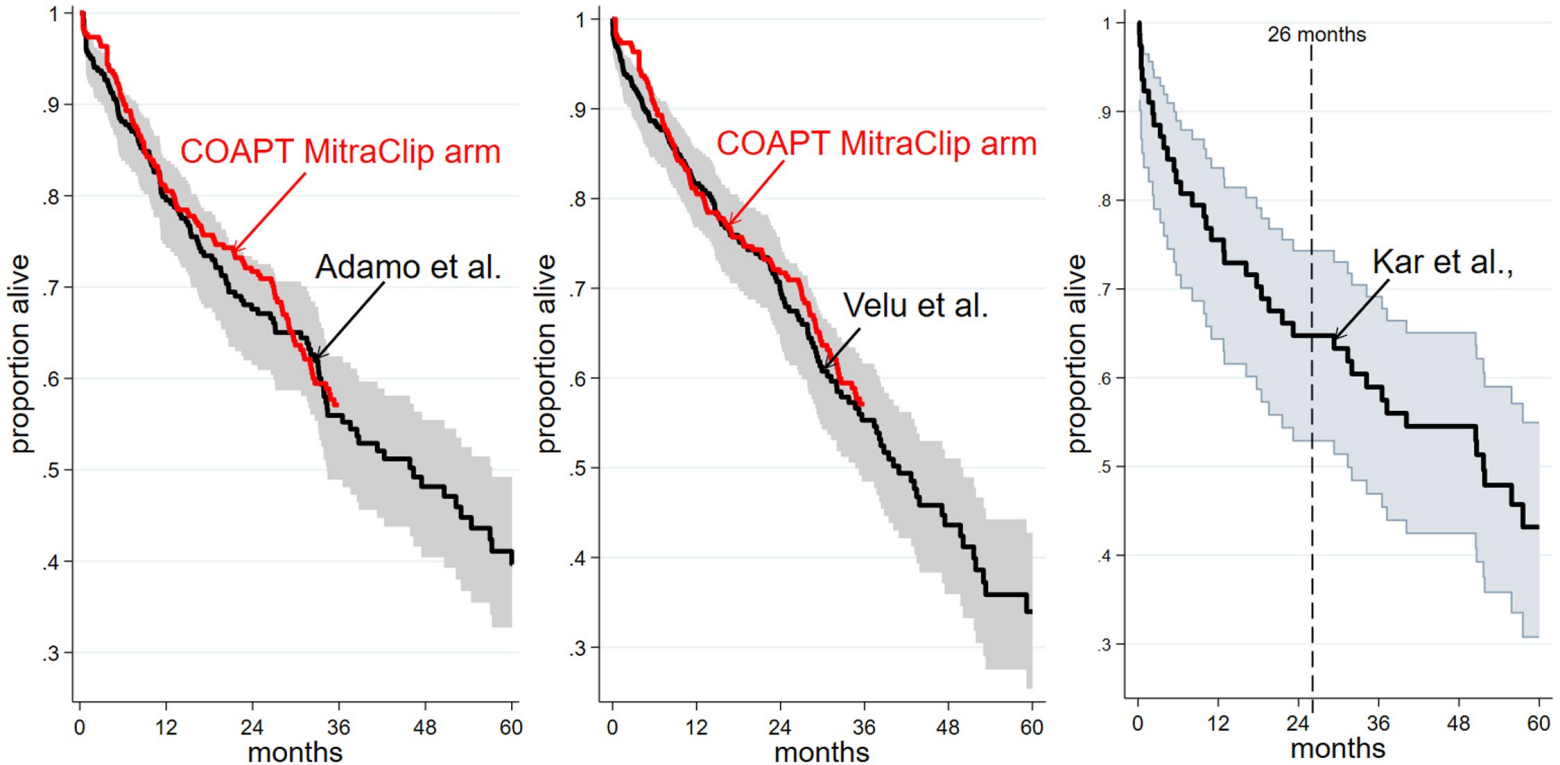
KM survival analysis of “real world” studies of PR with MitraClip compared to that for the PR + GDMT arm of COAPT. Red line represents the COAPT PR + GDMT KM, the black lines represent the reconstruted KM plots and 95% CI for “real world” studies.

The implied in-trial poorer survival in the PR + GDMT arm after two years could be attributable to a temporary anomaly, although this seems unlikely in view of the quality and size of COAT and the fact that half of participants were still at risk after 2 years follow up. We therefore looked for “real world” studies with substantial follow up beyond two years to see how these compared with the COAPT KM for the PR + GDMT arm followed for three years. We found two studies, Velu et al. 2017 [18] and Adamo et al. 2021 [19], with follow up to five years in populations comparable to that in COAPT (S2 Table in S2 File). Like the MitraClip arm in COAPT these KM plots indicate gradually decreasing slope to about 2.3 years followed by subsequent steeper trajectory (Fig 2). The steeper trajectory seen in these studies, particularly in Velu et al., aligns closely with that from COAPT (S2 Fig in S2 File). A further five year study (Kar et al. 2019 [20]) was identified in an older population carrying more co-morbidities than that in COAPT and at high risk from surgery (the EVEREST II HSR study [21]); like the other studies a gradually decreasing KM trajectory was seen to about 2 years followed by a steeper trajectory.

These studies suggest that the post-2 year survival downturn seen in the PR + GDMT arm of COAPT is not an exclusive feature of COAPT. We therefore compared observed three year COAPT survival with survival models developed in COAPT-based CE analyses to ascertain how previous CE studies’ modelling of survival might conform to or depart from the three-year in-trial findings. The various CE analyses have used several distinct and different approaches to model survival (S3 Table in S3 File).

**Fig 3:**
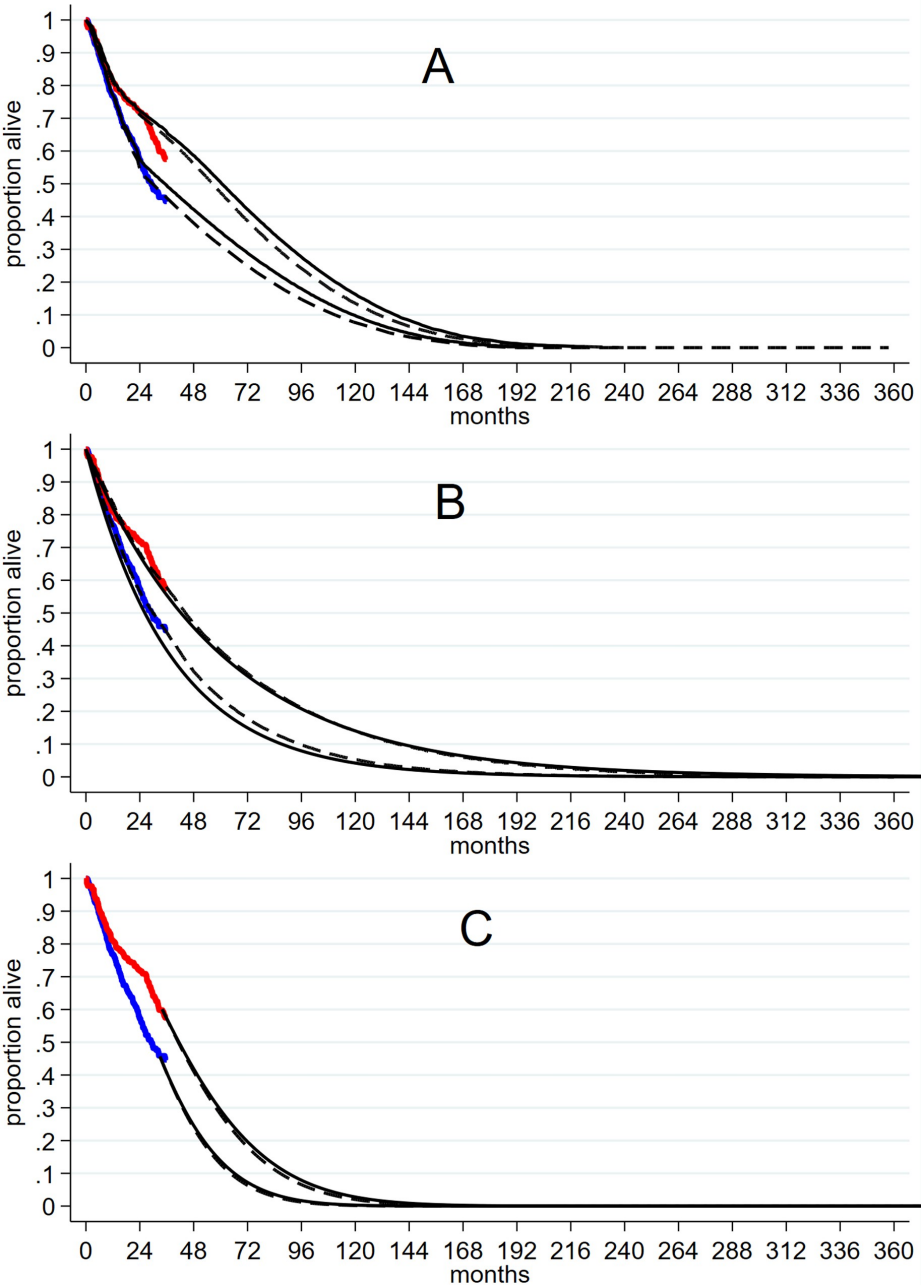
Survival models in CE studies compared to in-trial 3-year survival in COAPT. **A** 3-year COAPT in-trial survival (red intervention arm, blue GDMT arm) compared to Baron et al. (black dashed) and Cohen et al. (black solid) 2-year based survival models. **B** 3-year COAPT in-trial survival (red intervention arm, blue GDMT arm) compared to NICE guideline (dashed) and Shore (black solid) survival models. **C** 3-year COAPT in-trial survival (red intervention arm, blue GDMT arm) compared to Flexible parametric models with differing degrees of freedom (black solid and dashed).

Fig 3A and 3B show the models and extrapolations in these studies and compares them with three-year COAPT in-trial survival. The models of Baron and Cohen (Fig 3A) show a departure from the in-trial survival year 2 to year 3 that perpetuates in extrapolation. The models of Shore and NICE guideline (Fig 3B) conform poorly to the COAPT PR + GDMT KM from 2-year to 3-year follow up, although coinciding at three years. In extrapolation these two models seem divorced from the in-trial trajectory for the PR + GDMT arm. Shore and NICE guideline models for the GDMT arm differ slightly because one uses an exponential fit to 2-year data from COAPT and the other a Weibull fit to 3-year data.

**Fig 4:**
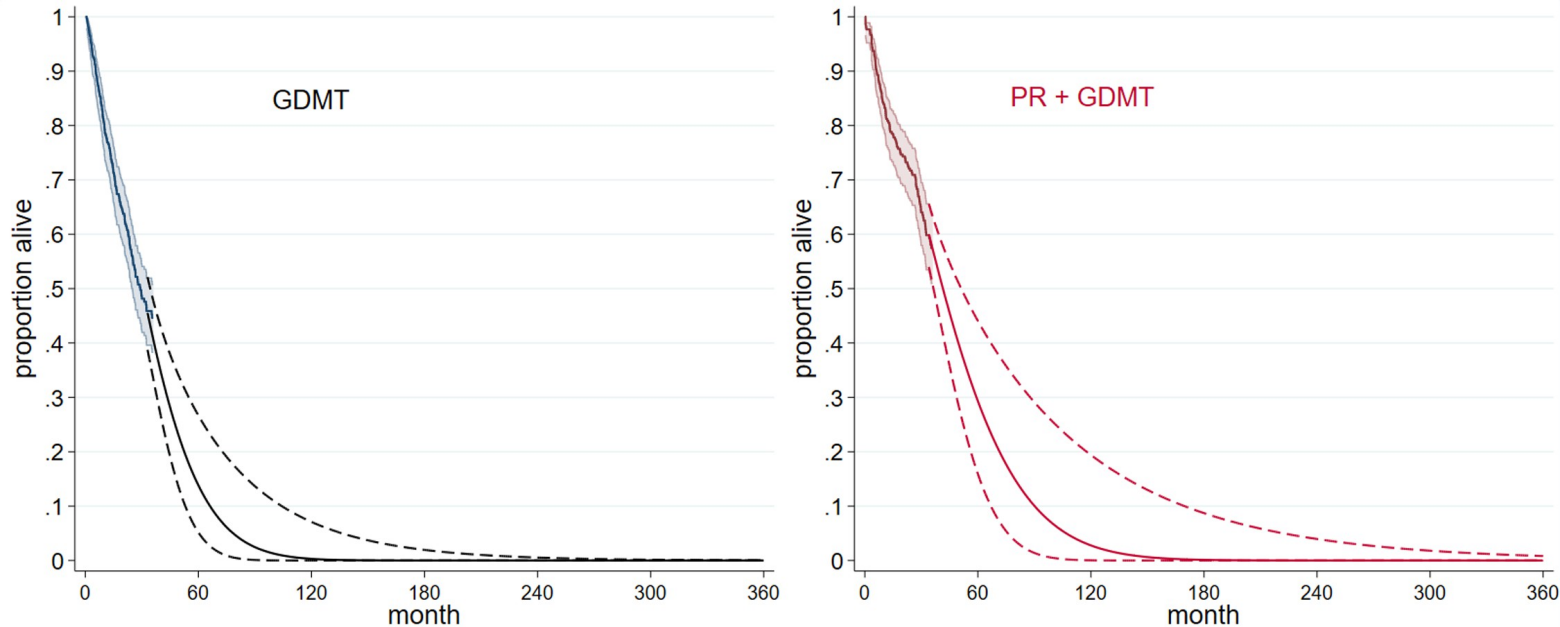
Flexible parametric survival models with 95% CIs used in the current cost-effectiveness analysis.

These comparisons indicate that previous CE models are likely to overestimate survival in the PR + GDMT arm when extrapolated beyond in-trial survival, and suggest that alternative modelling is required to capture the post-two year in-trial downturn in PR + GDMT survival. Standard parametric models (S4 Fig in S4 File and S5 Fig in S5 File) failed to generate good fit to 3-year in-trial survival particularly in the PR+GDMT arm whereas flexible parametric models provided good fit and accommodated the change in trajectory seen after 2-years (Figs 3C and 4).

### 3.2 Deterministic cost effectiveness analysis

Since standard parametric models failed to generate good fit to 3-year in-trial survival we employed a flexible parametric model that provided good fit and accommodated the change in trajectory seen after 2-years in the PR + GDMT arm.

We used in-trial survival to three years for both arms. Extrapolation beyond 3 years in the PR + GDMT arm used the flexible parametric model and 95% CIs; extrapolation beyond three years in the GDMT arm was obtained by applying the trial hazard ratio (0.67, Mack et al., 2021) to the intervention arm flexible model. The resulting models and extrapolations for each arm (Figs 3C and 4) had the advantages of good fit to observed data, plausible extrapolation beyond 3-year, consistency with the post 2-year survival downturn for PR + GDMT, and alignment with the real-world 5-year study of Velu et al., (S6A and S6B Figs in S6 File).

With 2.5% annual discounting (in line with a French perspective) this economic model generated ICERs of € 38,123 / QALY and € 25,416 / LYG, substantially greater than our previous analyses based on 2-year results from COAPT of € 21,918 / QALY and €14,434 / LYG. Estimates of cost effectiveness are seen to be strongly impacted by using updated data from COAPT. The current model delivers 3.6 LY and 2.7 LY in intervention and control arms respectively, providing an increment of 0.94 LY benefit.

**Fig 5:**
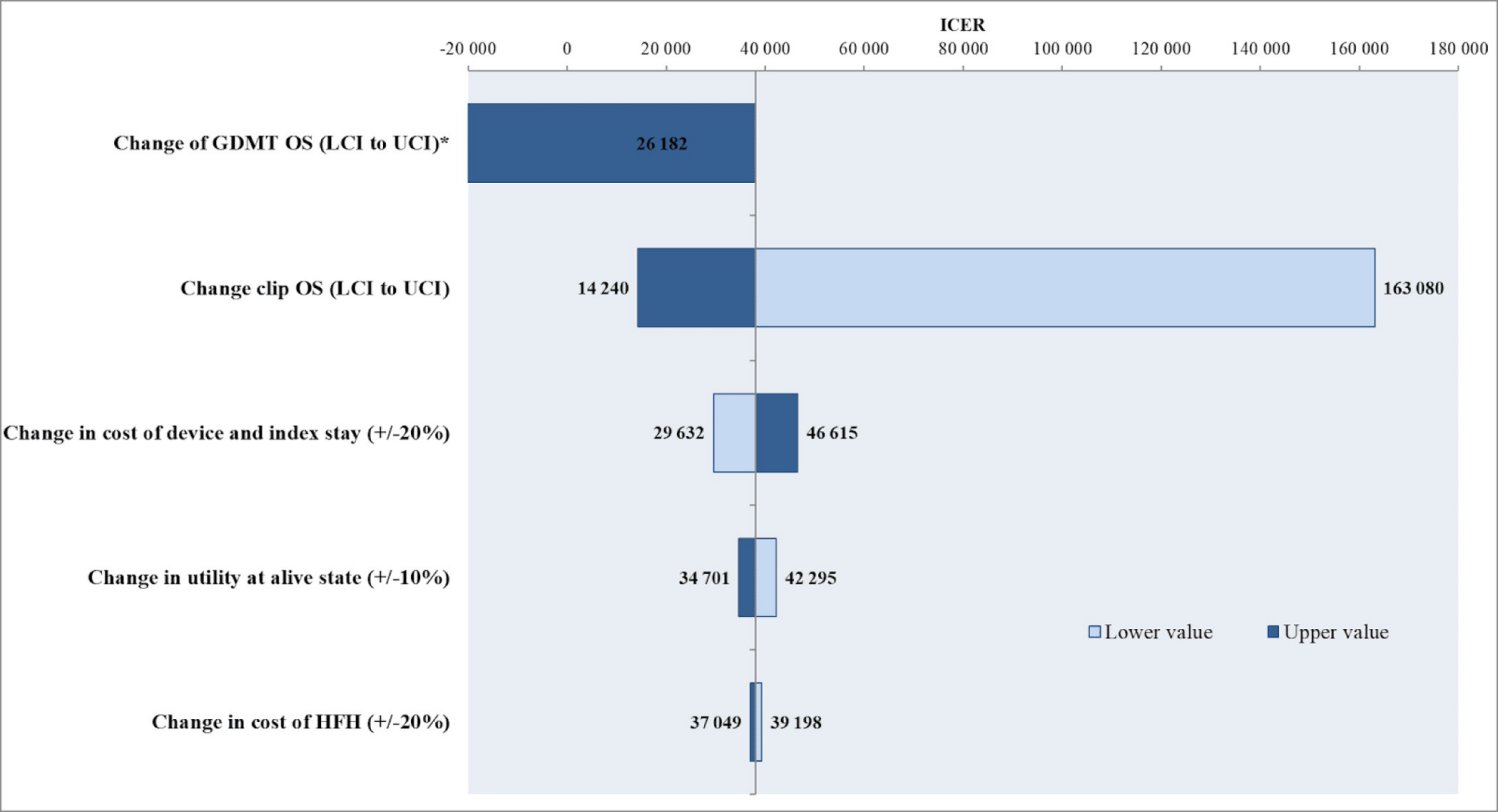
Tornado plot of univariate sensitivity analysis. * Using UCI value for OS in the GDMT arm, incremental effectiveness (Mitraclip relative to GDMT) becomes negative while incremental cost remains positive, resulting in a negative ICER (-7,550,815) denoting that the Mitraclip strategy is dominated; please note that the horizontal bar corresponding to the use of LCI value for OS in the GDMT arm (ICER of 26,182) is hidden below the one for UCI value for OS in the GDMT arm.

Fig 5 displays the results of univariate sensitivity analyses in the form of a Tornado diagram. The model was most sensitive to changing the survival input for each arm according to upper and lower 95% CIs. Other variables exerted relatively little effect on model output.

Lifetime life-year-gain (LYG) is an important element common to all the CE analyses, places our results in context and allows a direct comparison between different economic models avoiding complication from different jurisdiction costs and quality of life measures. Table 1 lists LY gains in previous CE studies and compares these with our results (using 3.5% annual discount to align with other studies). There is an approximate two-fold range across different studies highlighting the considerable influence of the different methodologies used for survival modelling and whether in-trial 2-year or in-trial 3-year COAPT mortality results are employed. Our estimate of incremental gain is appreciably less than that reported in all other CE studies other than Estler et al., and closest to those reported by Baron et al., and Estler et al.

**Table 1:** Estimates of lifetime LYG in CE analyses discounted at 3.5% annually^¥^.

Estimated life year benefit (annual discount at 3.5%)				
Study	LYG MitraClip + GDMT arm	LYG GDMT arm	Incremental LYG	% vs. lowest increment
Estler et al. [9]	3.68	2.88	0.80	100%
Cohen et al. [8]	Not reported	Not reported	1.57	196%
Baron et al. [7]	5.05	3.92	1.13	141%
Shore et al. [10]	4.56	3.01	1.55	194%
NICE guideline [11]*	Not reported	Not reported	1.44	148%
Current model§	2.61	3.50	0.89	111%
Armoiry et al. [6]	4.72	3.04	1.68	210%
Estimated quality-adjusted life year (QALY) benefit (annual discount at 3.5%)				
Study	QALY MitraClip + GDMT arm	QALY GDMT arm	Incremental QALY	% vs. lowest increment
Estler et al. [9]	2.50	1.93	0.57	100%
Cohen et al. [8]	4.31	3.19	1.12	196%
Baron et al. [7]	3.32	2.50	0.82	143%
Shore et al. [10]	3.06	1.98	1.07	188%
NICE guideline [11] *	2.92	2.05	0.87	152%

¥ the studies had various perspectives (e.g. US, UK, Germany).

* all previous studies except NICE used only two year in-trial results from COAPT

§ uses 3.5% annual discounting.

Because differing quality of life (utility) estimates have been employed in individual studies QALY benefit across studies may not directly relate to LY benefit, nevertheless despite considerable variation there is approximate correspondence between different benefit measures (Table 1).

We undertook scenario analyses to determine the effect on our LY benefit estimates of substituting our survival models with those from Baron, Shore or NICE-guideline (Table 2). The results tally reasonably well with those reported by the authors suggesting that most parameters in our current model do not differ radically from those in other CE analyses and indicate that the major difference between models is the method of modelling survival.

**Table 2:** Impact of other CE study’s survival models on ICER output from the current model.

Incremental benefit reported		Incremental benefit using current model with survival models from other studies	% difference in incremental benefit
Study	Incremental LYG	Incremental LYG	
Baron et al. [7]	1.13	1.22	7.9%
Shore et al. [10]	1.55	1.45	6.5%
Guideline [11]	1.44	1.22	16%
Current model	0.894	0.894	0%
Estimated QALY benefit			
Study	Incremental QALY	Incremental QALY	
Baron et al. [7]	0.82	0.81	1.3%
Shore et al. [10]	1.07	0.96	11%
Guideline [11]	0.87	0.81	7.4%
Current model	0.60	0.60	0%

### 3.3 Multivariate analysis

**Fig 6:**
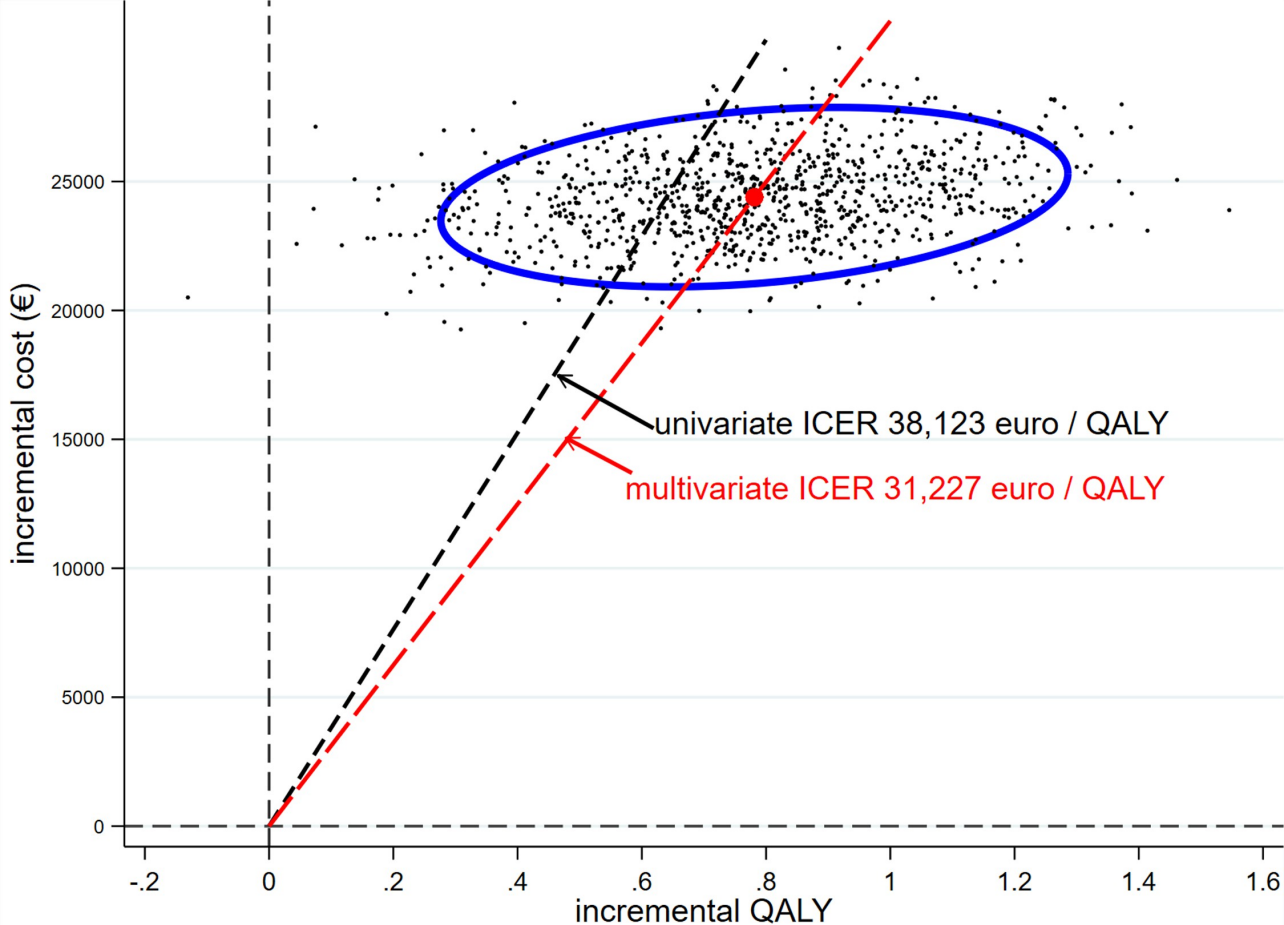
Univariate (black dashed line) and multivariate (red dashed line) results plotted on the cost-effectiveness plane. The black dots represent bootstrapped estimates, the blue elipse represents 95% CI around the mean multivariate bootstrap estimate.

The confidence intervals around survival models were wide (Fig 4); this together with uncertainty in costs and quality of life measures are likely to influence cost effectiveness estimates. We therefore conducted multivariate analysis varying survival according to 95% CI in each arm, major costs by ± 20% and out of hospital quality of life utility by ± 10%. Bootstrapping (Fig 6) produced a multivariate ICER estimate of € 31,227 / QALY, about 16% less than the deterministic ICER and more favourable to PR + GDMT. Both deterministic and multivariate estimates were very substantially greater than our previous estimate based on 2-year results from COAPT of € 21,918 /QALY.

## 4 Discussion

### 4.1 Survival after PR

Several statistical methods are available to extrapolate beyond the observed in-trial mortality data. These methods make use of different functional forms that, as is clear from previous CE analyses of PR (Table 1), can result major differences in estimated survival beyond the observed data. While such methods are valuable, especially when predicting costs and benefits over a protracted time horizon, their usefulness crucially hinges on the availability of rigorous and mature evidence [22]. If more mature evidence is consistent with earlier evidence then more mature data may tend to reduce the uncertainty in the clinical and CE estimates and offer a clearer picture of a technology’s long-term cost-effectiveness. In contrast if mature evidence is not consonant with earlier then a clearer picture is achieved with updating cost effectiveness analysis. In the present case we conclude that mature evidence from COAPT is somewhat inconsistent with the two-year trial data and that this conclusion is supported by “real world” evidence from other studies. Therefore updated cost-effectiveness analysis is necessary.

### 4.2 Cost-effectiveness based on mature trial data and previous economic analyses

It seems axiomatic that cost-effectiveness (CE) estimates are best served by making use of the most mature trial data available. Our analyses is the first to contribute this new perspective for PR in that we take account of the downturn in survival seen in the MitraClip arm of COAPT after two years, for proper comparison earlier CE studies require update. Should the downturn transpire to be anomalous in light of longer term results from COAPT (four year results from COAPT are awaited), or are very particular to only the COAPT population then our perspective can be replaced by further updated CE estimatation based on later COAPT trial findings. The available “real world” studies [18–20] with follow up to 5 years support the proposition that the survival downturn is not an anomaly.

The two-year results fom COAPT [2] have been used in at least six published cost-effectiveness analyses [5–10], all these, including our own, indicating that PR with MitraClip is likely to be cost-effective relative to commonly employed willingness-to-pay thresholds in various jurisdictions. Our current analysis based on three-year follow-up findings from COAPT that first became available in September 2019 [3] indicate that earlier estimates based on two-year data from COAPT may deliver ICERs underestimated by about 30%. Four-year COAPT results, taking follow-up data to 2019, were expected in 2020 but at time of writing (December 2022) have not yet reached the public domain; when available 4-year findings should be used to update CE estimates. Five-year follow-up COAPT results will be complicated by the arrival of COVID-19, these will obviously be of intrinsic interest but will be difficult to incorporate into CE analysis of PR.

It could be argued that the GDMT arm might exhibit a downturn in survival similar to that seen for the MitraClip arm after two years follow up if cross-over to MitraClip after two years had not been permitted for GDMT recipients. At two years, 144 patients remained at risk but only 53 subsequently crossed over. KM plots for the GDMT arm based on two and three-year follow up were very similar over the first two years with the further year follow up continuing the similar trajectory (S7 Fig in S7 File) suggesting that cross over occurring late between two and three years had very little beneficial effect within the three years of follow-up. For the crossovers to have a significant effect on survival in the period from two to three years they would require to have coincidentally been received for very ill patients that would have otherwise died and generated a downturn like that seen for the intervention arm. This seems unlikely in practice since mortality risk of PR is greatest in the thirty days post-intervention. Most of the post two-year crossovers occurred late in the period two to three years [3, 4] so a rapid influence of cross-over would additionally be necessary to change the observed in-trial survival; the trajectory of the OS GDMT KM plot remains very stable and there is no major indication of perturbation in the trajectory of the plot. Methods are available for adjusting survival estimates for cross-over; these require individual patient data not in the public domain. There is insufficient data in the pubic domain to adjust cumulative HFH in the GDMT arm for cross over from GDMT to MitraClip + GDMT, however since the ITT analysis including cross-overs exhibits an increased rate between 2 and 3 years it appears unlikely cross-over confers and advantage to the GDMT arm with regard to cumulative HFH that could lead to lower ICERs.

There are several limitations to our analyses. We used reconstructed individual patient-level data (IPD) rather than the patient data from the COAPT trial; however due to the high quality of the plots available from Mack et al., [3] and the use of robust methods [15] we believe our KM survival plots are very closely similar to those published. We employed parametric model extrapolations beyond the observed survival data, a widely employed procedure in cost-effectiveness analysis which, although unavoidable for lifetime analysis, must inevitably always be open to question, even though the model choice may be informed by visual fit, statistical criteria and clinical plausibility. The use of three health states here, as elsewhere, represents an oversimplification of the experience of patients with MR but was a necessary limitation due to lack of additional three year information. We explored uncertainty in ICERs by bootstrapping rather than using probabilistic analysis. Lastly, based on available data, we used the same utility inputs throughout the entire model duration.

### 4.3 Implications

Our work has clear implications: in this study the availability of updated survival analyses of the main trial is likely to have relevance to decision-making and/or pricing discussion as part of health-technology assessment (HTA). Indeed, should a health technology have a less or a more favourable ICER with more mature survival data, one would expect that any corresponding patient access scheme might be revised accordingly. There is however no implication of our work at physician level since our results cannot be used for the purpose of patient selection.

More broadly, technology appraisals are frequently undertaken when mid/long-term follow-up trial data may be lacking. This was recently emphasized by Tai et al. [23] based on a review of NICE decisions for cancer drugs. These authors reported that the use of immature survival data to inform reimbursement decisions made by NICE was as high as 41%. These data suggest the need for continuous HTA review when more mature clinical data are released and additional comparator treatments become approved.

Another contribution of our work is that the elements of model structure we employed are simple and adaptable, and have the potential to be readily populated with data pertaining to devices for additional cardio-vascular conditions.

## 5 Conclusion

Analyses of the cost-effectiveness of the MitraClip device should be updated in the light of the latest available data. Our results indicate that the difference in observed survival of MitraClip recipients between two-year results and three-year results from COAPT will appreciably influence cost-effectiveness estimates.

## Supporting information

S1 File Cumulative mortality in COAPT at 3 years. Red plot = PR + GDMT, black plot = GDMT.
(PDF)

S2 File COAPT and real world studies. Table of demographic characteristics of real world studies and of COAPT. Figure with COAPT at three years (green) versus real world studies (red = Adamo et al., black = Velu et al.).
(PDF)

S3 File Modelling of survival in economic anlyses of PC repair using MitraClip. Table with previous studies.
(PDF)

S4 File Standard parametric models of PR + GDMT arm of COAPT. Figure with Standard parametric models (red lines) were fit to in-trial survival for the GDMT arm of COAP (black line with 95% CI).
(PDF)

S5 File Extrapolation of standard parametric models. Figure showing Standard parametric models that were fit independently to in-trial survival (PR + GDMT red, GDMT black) arms of COAPT an-d extrapolated to 20 years.
(PDF)

S6 File Flexible parametric modelling of overall survival in the PR + GDMT arm of COAPT 3 YEAR DATA. Fig A: Flexible parametric model (red line) and 95% CI (blue line) 3-yr follow up; Fig B: Modelled extrapolation beyond 3-yr follow up COAPT (green line) compared to five year real world study of Velu et al.
(PDF)

S7 File GDMT overall survival in COAPT. Figure with the Comparison of two year (black) and three year (red) Kaplan Meier analyses.
(PDF)
