# Supplementary material for: Impact of updated trial data on the cost-effectiveness of percutaneous mitral repair
Source: PLoS One. 2023 Jan 26;18(1):e0280554. doi: 10.1371/journal.pone.0280554 (PMC9879464; doi:10.1371/journal.pone.0280554)

SUPPLEMENTARY MATERIAL S 1

Cumulative mortality in COAPT 3 year analysis

S1 Figure Red plot = PR + GDMT, Black plot = GDMT

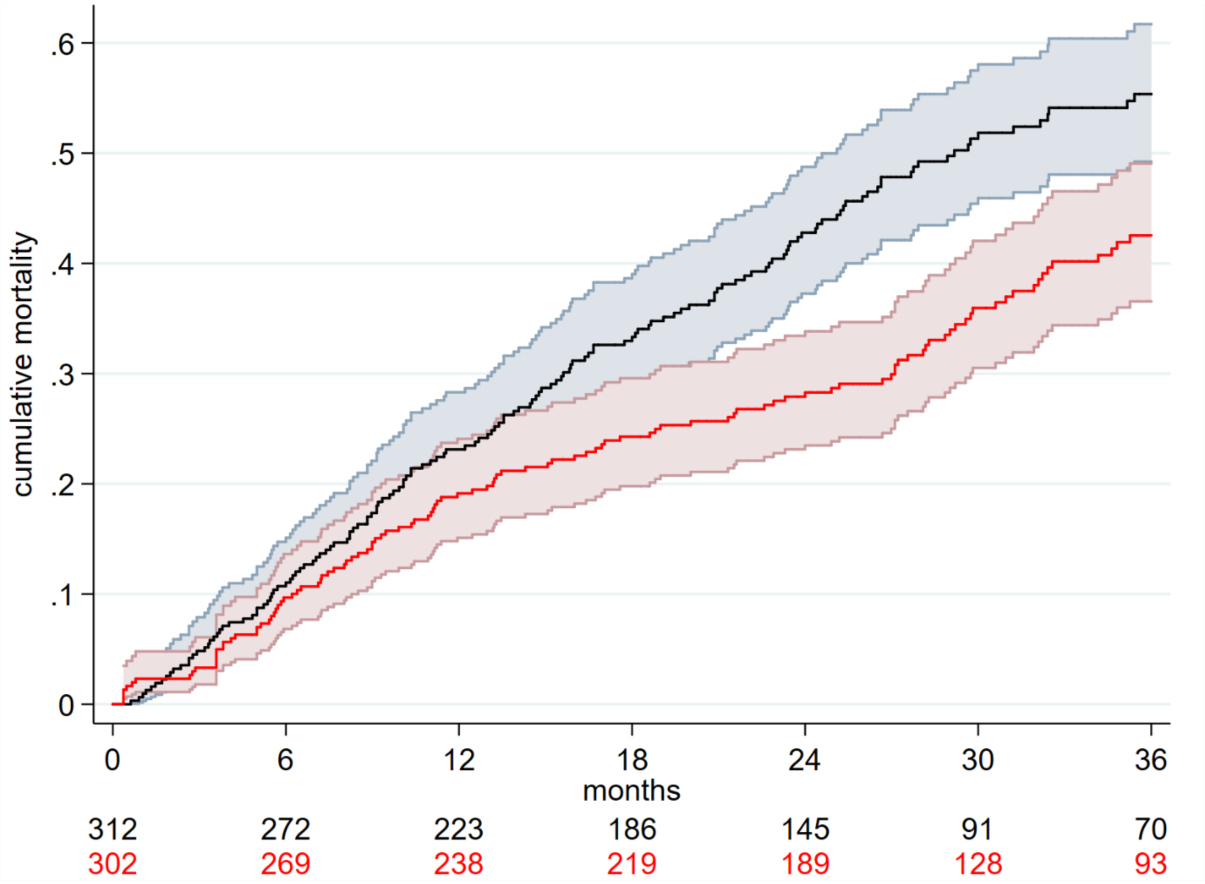

Supplement: S1 File — Red plot = PR + GDMT, black plot = GDMT. (PDF) [file pone.0280554.s001.pdf]
