# Supplementary material for: Impact of updated trial data on the cost-effectiveness of percutaneous mitral repair
Source: PLoS One. 2023 Jan 26;18(1):e0280554. doi: 10.1371/journal.pone.0280554 (PMC9879464; doi:10.1371/journal.pone.0280554)

SUPPLEMENTARY MATERIAL S2

S2 COAPT and real world studies

S2 TABLE Demographic characteristics of real world studies and COAPT

	**COAPT PR+GDMT (N=302)**		**Adamo (N=304)**		**VELU (N=326)**		**KAR (N=78)**	
	**value**	**%**	**value**	**%**	**value**	**%**	**value**	**%**
Age yr	71.7		71.5		76		76.7	
Male # %	201	66.6	225	74.0	185	57		62.8
Diabetes # %	106	35.1	103	33.9	69	21		41
Hypertension # %	243	80.5	183	60.2	176	54		
Hypercholesterolemia # %	166	55						
Previous MI # %	156	51.7	156	51.32				55.8
Previous percutaneous intervention # %	130	43	123	40.46	88	27		
Previous CABG # %	121	40.1	76	25	94	29		62.8*
Previous stroke or TIA # %	56	18.5	14	4.61	33	10		
Peripheral VD # %	52	17.2	43**	14.15				18.2
Chronic obstructive PD # %	71	23.5	55	18.1	64	20		34.6
History of atrial fibrillation or flutter # %	173	57.3	117	38.5		100		61.6
Body-mass index kg/m^2	27.0		30					
Creatinine clearance Mean ml/min‡	50.9				54[¥]			
Creatinine ≤60 ml/min‡	*214/299*	*71.6*	***218***	***71.7***[§]				
STS risk score Mean %	*7.8*		***6.4*** [¥¥]		*19.8* [ΔΔ]			
STS ≥ 8% # %	126	41.7						
NYHA class n/N; %							Data Whitlow[π]	
I	1/302	0.3				0	0/54	0
II	129/302	42.7	52/304	17.1		12	1/54	1.9
III	154/302	51	180/304	59.2		70	38/54	70
IV	18/302	6 [ππ]	72/304	23.7		18	17/54	31.5
Hospitalization for HF in previous yr # %	176	58.3	191	62.8				
Previous resynchronisation therapy # %	115	38.1	115	37.9	98 [∞∞]	30		35.1[§§]
Previous implantation of defibrillator # %	91	30.1						
B-type natriuretic peptide level ng/L	1014.8		763.9					
N-terml pro–B-type natriuretic peptide level ng/L	5174.3		1363.4	1905				
Severity of mitral regurgitation								
Moderate-to-severe, grade 3+	*148*	*49*						
Severe, grade 4+	*154/*	*51*			203[««]	62		

* Previous cardiovascular surgery; ** peripheral arterial disease; ¥ glomerular filtration rate ml/min/1.73m^2; § chronic kidney disease; ¥¥EuroSCORE II %; ΔΔ LESCORE (%);π 56 patients listed but 54 quoted; ππ ambulatory; ∞∞ Cardiac implantable electronic devices; §§ Pacemaker or ICD implant; «« Mitral regurgitation grade 4

S2 FIGURE

COAPT at three years (green) versus real world studies (red = Adamo et al., black = Velu et al.)

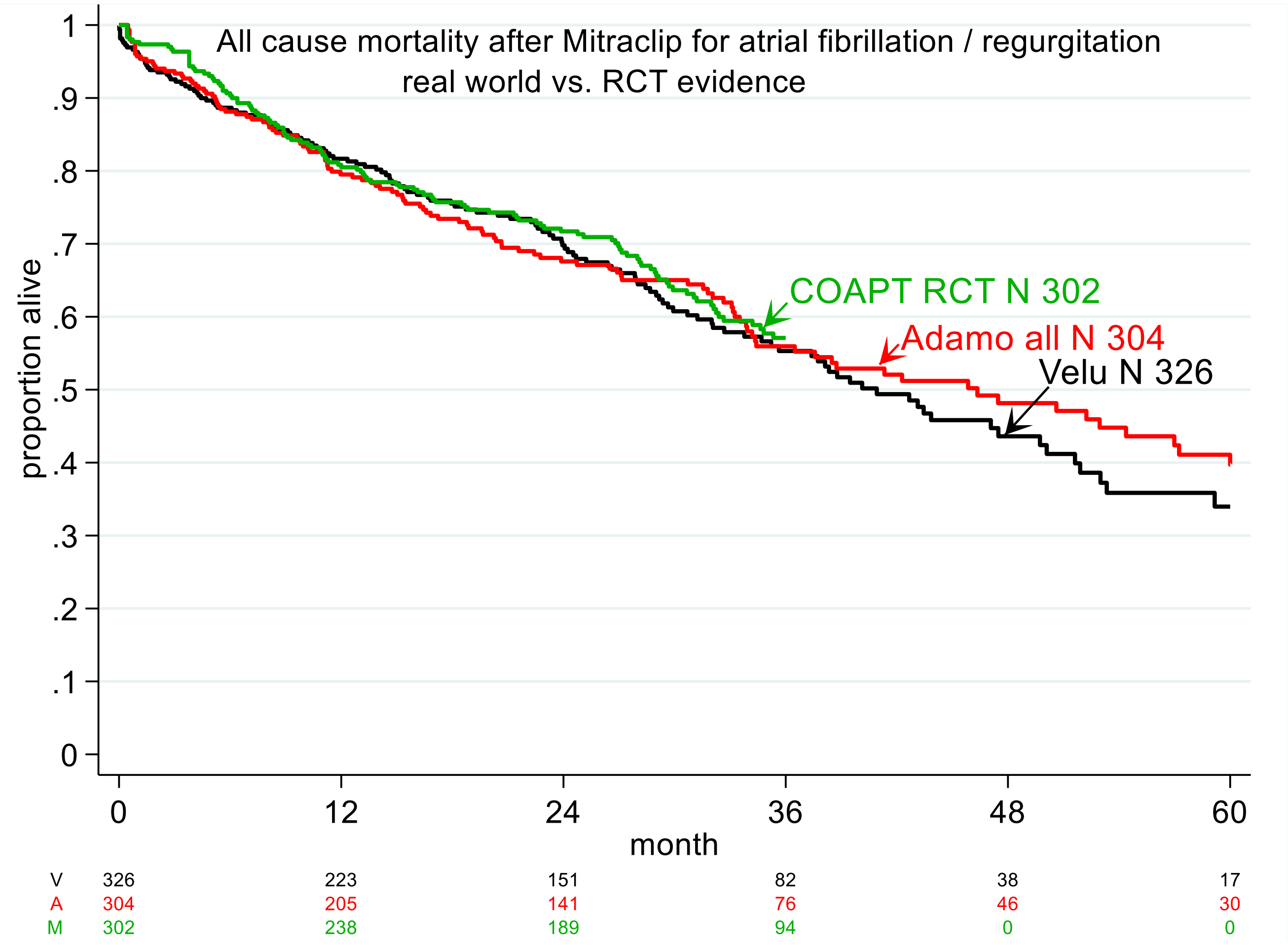

Supplement: S2 File — Table of demographic characteristics of real world studies and of COAPT. Figure with COAPT at three years (green) versus real world studies (red = Adamo et al., black = Velu et al.). (PDF) [file pone.0280554.s002.pdf]
