# Supplementary material for: Impact of updated trial data on the cost-effectiveness of percutaneous mitral repair
Source: PLoS One. 2023 Jan 26;18(1):e0280554. doi: 10.1371/journal.pone.0280554 (PMC9879464; doi:10.1371/journal.pone.0280554)
Supplement: S3 File — Table with previous studies. (PDF) [file pone.0280554.s003.pdf]

SUPPLEMENTARY MATERIAL S3

S3 **Modelling of survival in economic analyses of PC repair using MitraClip**

S3 Table Previous studies

**Baron et al.,** used 2-year patient level data from COAPT, adopted the KM plots to 2 years from Stone et al. Modelling was accomplished using *"in-trial data … to project patient-level survival over a lifetime perspective; and uncertainty was assessed with bootstrap resampling"*. Projected survival beyond the in-trial 2-year KM was estimated separately for the TMVr (PR + GDMT) and GDMT groups. For the GDMT group, survival between time of randomization and last observed follow-up within 2 years was compared with expected age- and sex-adjusted mortality using US life tables to calculate a calibration factor (relative mortality hazard). For each surviving patient, life expectancy beyond the last observed follow-up was then estimated from recalibrated life tables. Survival for the TMVr group (PR + GDMT) was estimated in an analogous fashion after application of the hazard ratio for mortality after TMVr versus GDMT derived from a landmark analysis of trial data between 30 days and the last observed follow-up within 2 years. This landmark analysis was chosen to minimize the effect of peri-procedural complications on the long-term"
**Cohen et al.,** used Barron's procedure adapted to life tables for UK patients.<br>**In summary** Baron and Cohen modelled mortality separately for each arm, used 2 yr COAPT data that lacks the observed post 2-year downturn in survival in the intervention arm.
**Shore et al., and NICE guideline** modelled the GDMT arm (that has no post 2-year downturn) with exponential or Weibull parametric models respectively and applied a HR to obtain a MitraClip arm model that consequently will not take into account the observed downturn in that arm post two-years. Shore employed COAPT 2-year GDMT survival and HR, while the NICE guideline used COAPT 3-year GDMT survival and HR.<br>**In summary** Shore and NICE guideline did not model arms separately and emplyed parametric fit with HR.
**Estler et al.** Modelled survival differently to the studies of Cohen et al. , Baron et al , and Shore et al, but like these also derive survival data from the COAPT 2-year follow up findings rather than 3-year. In the Estler study, modelled mortality depends on the evolution of different NYHA classes through time (Estler et al, Table 1), with only NYHA III and IV classes allocated a transition probability to death > than zero. Thus the time changing proportion of patients in these two NYHA classes governs mortality and these proportions in turn are determined by the transition probabilities (TP) applied for transitions between different NYHA classes (see Estler et al., model diagram Fig 1 and TPs listed in Table 1). The author's request for these transition data from COAPT was unsuccessful and so an approximation was assumed. One way sensitivity analyses (see Estler et al Tornado diagram in Supplementary material) demonstrated that model output was highly sensitive to change in the TP from class II to III and from III to IV (i.e. to those classes with a death TP > zero).**In summary** a novel procedure was used that does not take into account the post two-year downturn in survival in the intervention arm.
**Armoiry et al.,** modelled survival in essentially the same way as Shore et al. based on 2-year findings from COAPT. This procedure does not take into account the post two-year downturn in survival in the intervention arm.
