# Supplementary material for: Impact of updated trial data on the cost-effectiveness of percutaneous mitral repair
Source: PLoS One. 2023 Jan 26;18(1):e0280554. doi: 10.1371/journal.pone.0280554 (PMC9879464; doi:10.1371/journal.pone.0280554)

**SUPPLEMENTARY MATERIAL S4**

**S4 Standard parametric models of PR + GDMT arm of COAPT**

S4 Figure Standard parametric models (red lines) were fit to in-trial survival for the PR + GDMT arm of COAPT (black line with 95% CI).

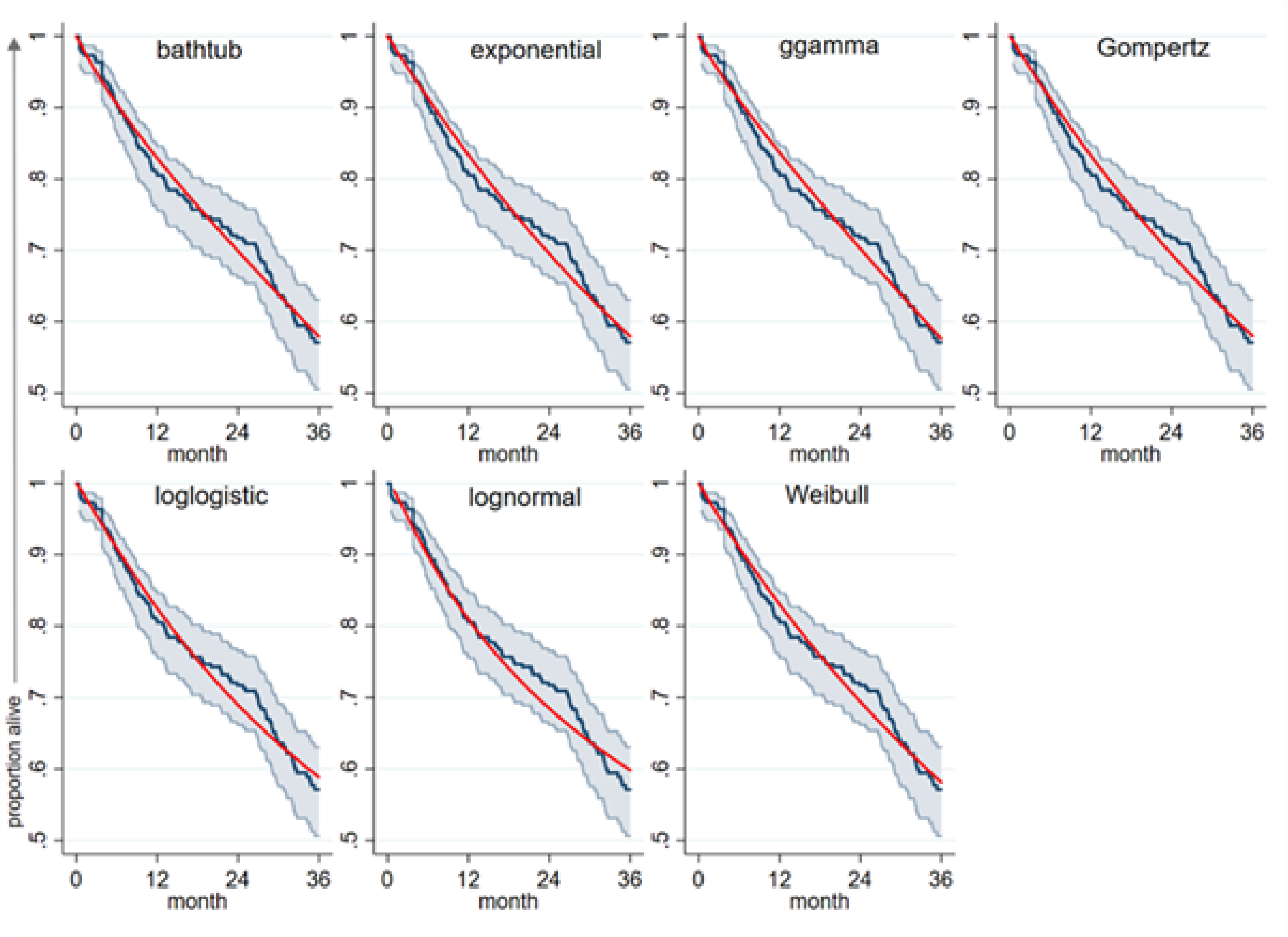

Supplement: S4 File — Figure with Standard parametric models (red lines) were fit to in-trial survival for the GDMT arm of COAP (black line with 95% CI). (PDF) [file pone.0280554.s004.pdf]
