# Supplementary material for: Impact of updated trial data on the cost-effectiveness of percutaneous mitral repair
Source: PLoS One. 2023 Jan 26;18(1):e0280554. doi: 10.1371/journal.pone.0280554 (PMC9879464; doi:10.1371/journal.pone.0280554)

SUPPLEMENTARY MATERIAL S 5

S5 Extrapolation of standard parametric models

S5 Figure Standard parametric models were fit independently to in-trial survival for the PR + GDMT (red) and GDMT (black) arms of COAPT and extrapolated to 20 years.

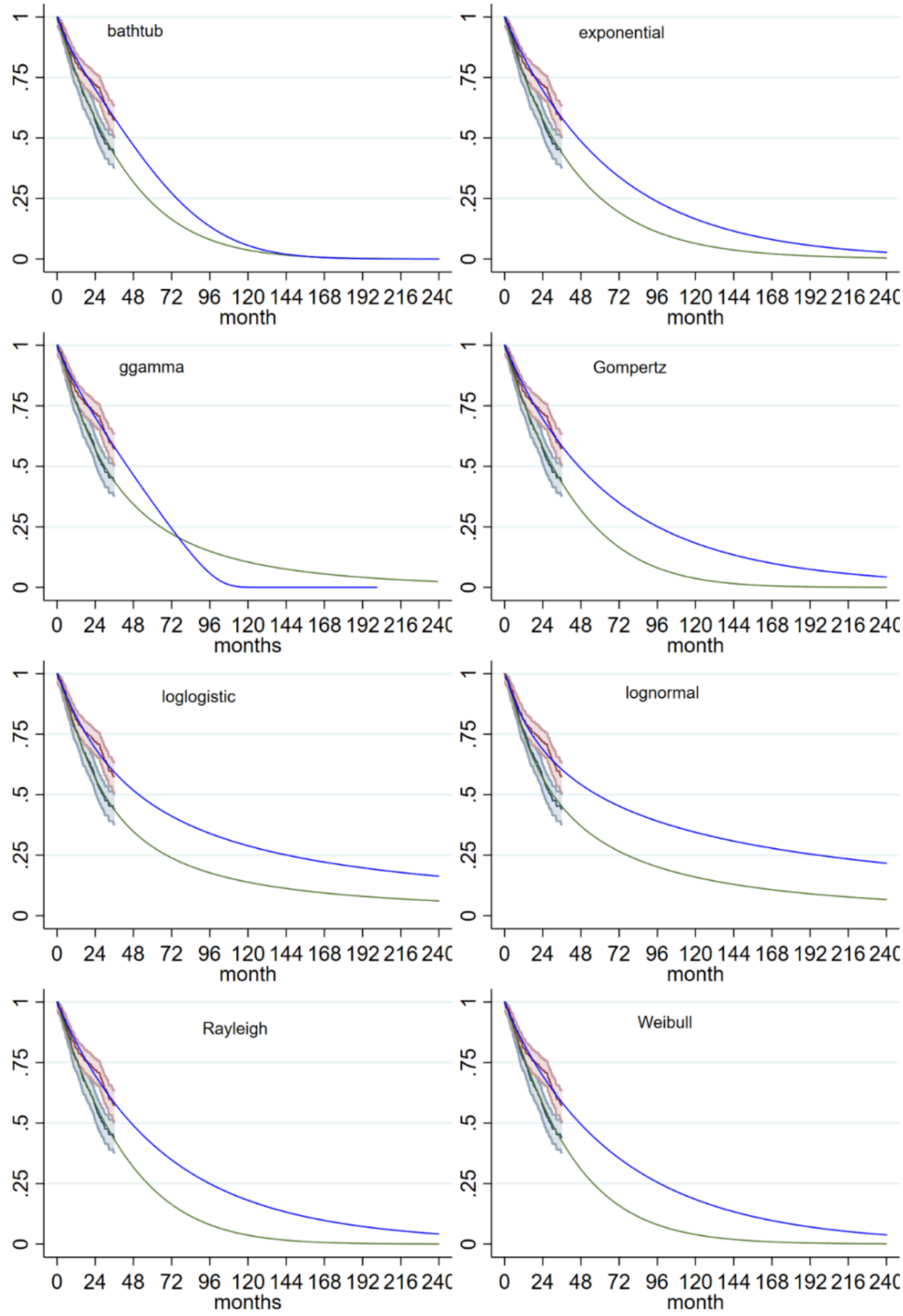

Supplement: S5 File — Figure showing Standard parametric models that were fit independently to in-trial survival (PR + GDMT red, GDMT black) arms of COAPT an-d extrapolated to 20 years. (PDF) [file pone.0280554.s005.pdf]
