# Supplementary material for: Impact of updated trial data on the cost-effectiveness of percutaneous mitral repair
Source: PLoS One. 2023 Jan 26;18(1):e0280554. doi: 10.1371/journal.pone.0280554 (PMC9879464; doi:10.1371/journal.pone.0280554)

SUPPLEMENTARY MATERIAL S 6

Flexible parametric modelling of overall survival in the PR + GDMT arm of COAPT 3 YEAR DATA

Fig S6 A: Flexible parametric model (red line) and 95% CI (blue line) 3-yr follow up; B: Modelled extrapolation beyond 3-yr follow up COAPT (green line) compared to five year real world study of Velu et al.

A

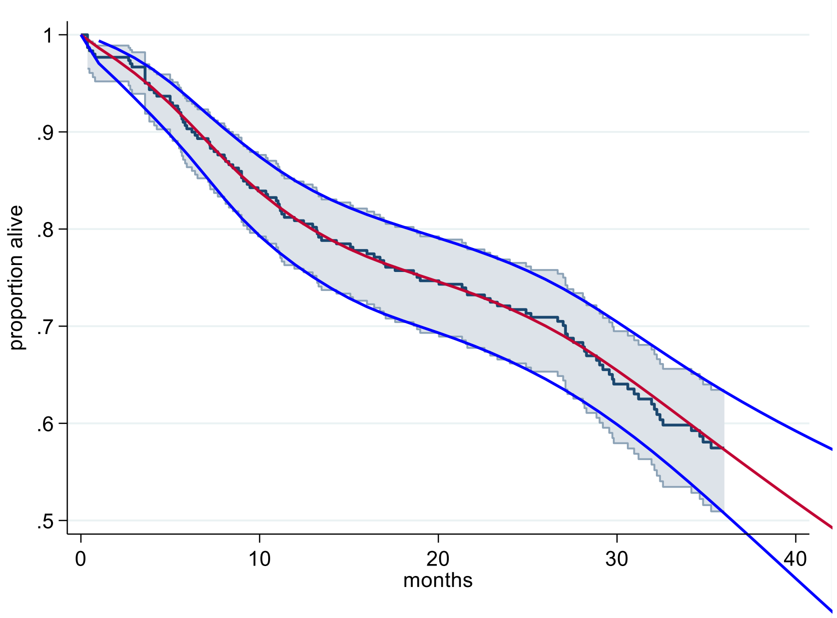

B

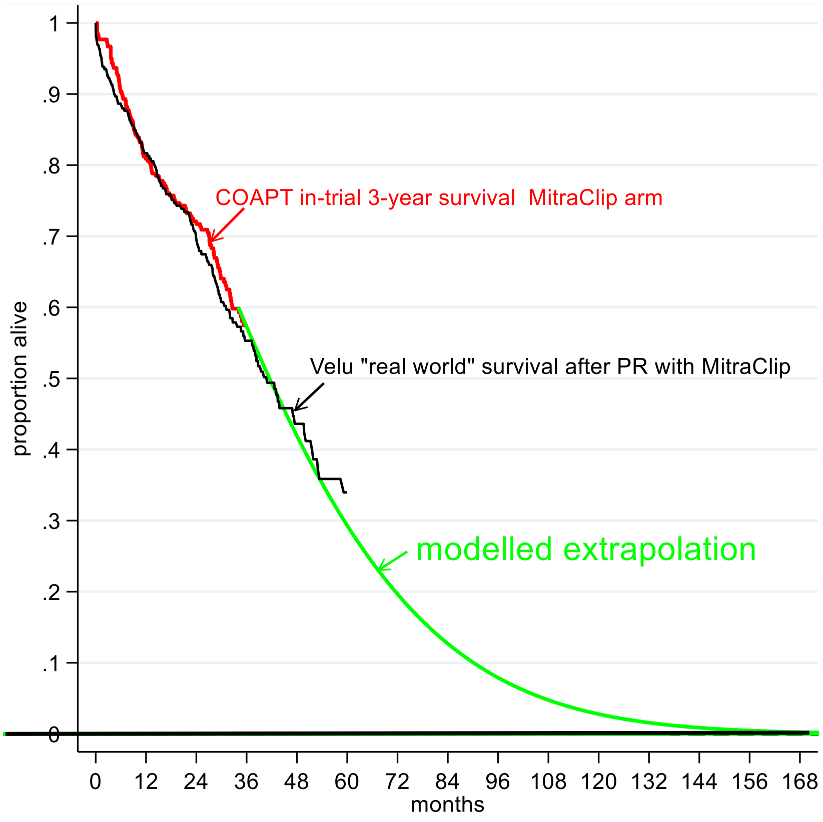

Supplement: S6 File — Fig A: Flexible parametric model (red line) and 95% CI (blue line) 3-yr follow up; Fig B: Modelled extrapolation beyond 3-yr follow up COAPT (green line) compared to five year real world study of Velu et al. (PDF) [file pone.0280554.s006.pdf]
