# Supplementary material for: Impact of updated trial data on the cost-effectiveness of percutaneous mitral repair
Source: PLoS One. 2023 Jan 26;18(1):e0280554. doi: 10.1371/journal.pone.0280554 (PMC9879464; doi:10.1371/journal.pone.0280554)

SUPPLEMENTARY MATERIAL S 7
GDMT overall survival in COAPT
S7 Figure Comparison of two year (black) and three year (red) Kaplan Meier analyses

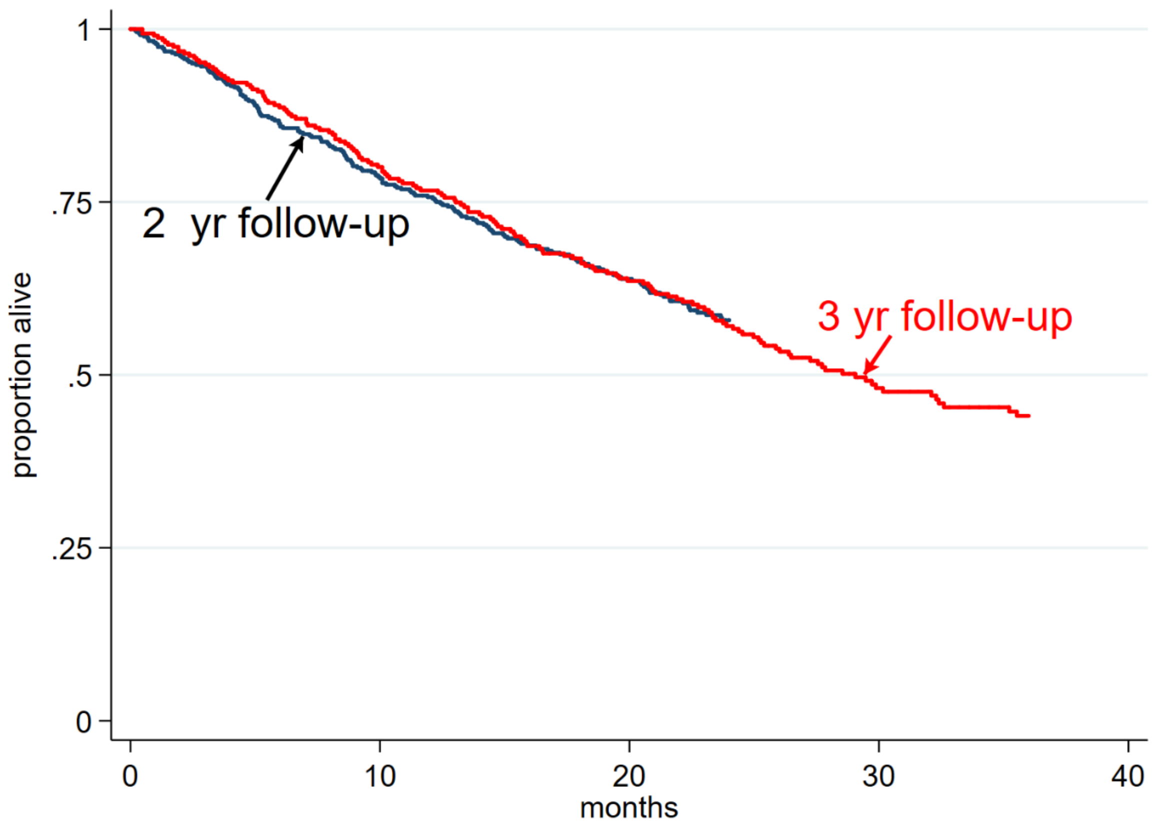

Supplement: S7 File — Figure with the Comparison of two year (black) and three year (red) Kaplan Meier analyses. (PDF) [file pone.0280554.s007.pdf]
